# Childhood Maltreatment, Mental Well-Being, and Healthy Lifestyle in Patients With Chronic Thromboembolic Pulmonary Hypertension

**DOI:** 10.3389/fpsyt.2022.821468

**Published:** 2022-02-24

**Authors:** Nicole Lepsy, Madelaine-Rachel Dering, Jan Fuge, Tanja Meltendorf, Marius M. Hoeper, Ivo Heitland, Jan C. Kamp, Da-Hee Park, Manuel J. Richter, Henning Gall, Hossein A. Ghofrani, Dietmar Ellermeier, Hans-Dieter Kulla, Karen M. Olsson, Kai G. Kahl

**Affiliations:** ^1^Department of Psychiatry, Social Psychiatry and Psychotherapy, Hannover Medical School, Hannover, Germany; ^2^Department of Respiratory Medicine, Hannover Medical School, German Center for Lung Research (DZL/BREATH), Hannover, Germany; ^3^Department of Internal Medicine, German Center for Lung Research (DZL), Justus Liebig University Giessen, Universities of Gießen and Marburg Lung Center, Giessen, Germany; ^4^Department of Pneumology, Kerckhoff Heart, Rheuma and Thoracic Center, German Center for Lung Research, Universities of Giessen and Marburg Lung Center, Bad Nauheim, Germany; ^5^Pulmonale Hypertonie Selbsthilfe, Bottrop, Germany; ^6^Pulmonale Hypertonie eV, Rheinstetten, Germany

**Keywords:** chronic thromboembolic pulmonary hypertension, pulmonary hypertension, childhood maltreatment, childhood trauma questionnaire, CTQ, CTEPH, quality of life

## Abstract

**Introduction:**

Chronic thromboembolic pulmonary hypertension (CTEPH) is a potentially life-threatening condition associated with high morbidity and mortality. However, advances in medical, surgical and interventional treatment have markedly improved the outcome of patients with CTEPH. Additional factors potentially influencing quality of life (QoL) and outcome in CTEPH are yet to be defined. Child maltreatment is a major risk factor for unfavorable behavioral, mental as well as physical health outcomes and has been associated with decreased QoL. To date, no study assessed the impact of childhood trauma in patients with CTEPH.

**Methods:**

Patients with CTEPH were invited to complete the Childhood Trauma Questionnaire (CTQ). Data were compared to prevalence data from the German population. Mental well-being was assessed using the Hospital Anxiety and Depression Scale (HADS) and quality of life was measured using the WHO Quality of Life Questionnaire (WHOQOL). Furthermore, lifestyle factors and physical health parameters were studied.

Logistic regression analysis was used to investigate a possible impact of child maltreatment on markers of disease severity.

**Results:**

One-hundred and seven patients with CTEPH completed the CTQ. These patients reported higher rates of emotional abuse and physical abuse and emotional neglect compared to the German population while rates of physical neglect and sexual abuse did not differ between patients and German population with prevalence of 20.6% for emotional abuse, 20% for physical abuse, 22% for emotional neglect, 46% for physical neglect, and 6% for sexual abuse in patients with CTEPH. Higher CTQ scores were associated with anxiety symptoms as well as negatively associated with QoL. No direct impact of childhood trauma on CTEPH severity was found.

**Conclusion:**

We found a higher rate of child maltreatment in patients with CTEPH in comparison to the German population. Correlations suggest moderate associations between CTQ scores and mental health and QoL. Child maltreatment had no significant impact on disease severity. Further investigation on proper interventions to support affected patients is needed.

## Introduction

Chronic thromboembolic pulmonary hypertension (CTEPH) is a chronic disease occurring as a possible complication after pulmonary embolism. There are several entities of pulmonary hypertension (PH), categorized into five groups by the World Health Organization (WHO). Of these, CTEPH is designated as WHO group 4.1 (1). In CTEPH, unresolved thromboembolic material leads to the occlusion of pulmonary arteries and progressive pulmonary vascular remodeling resulting in elevated pressure and resistance in the pulmonary vascular bed (2). As a consequence, patients are initially experiencing rather unspecific symptoms like progressive dyspnea on exertion and fatigue as well as syncope and signs of right heart failure in advanced disease (3). With pulmonary endarterectomy (PEA) as surgical treatment, CTEPH became a potentially curable disease for operable patients. The 3-year survival rate increased from estimated 40–90% in patients who underwent surgery. While the majority of operable patients can be cured or achieve functional improvement, residual or recurrent CTEPH might be present (4, 5). Further on, about one half of patients with CTEPH is considered inoperable and therefore receiving lifelong medical treatment and interventional therapy if possible (6, 7). Despite improvements in treatment and increased life expectancy, quality of life is impaired in comparison to the general population (8, 9), and concomitant mental disorders have shown to be associated with impaired physical health (10). Taking these results into account, other factors, increasing the risk of impaired quality of life, physical health and developing mental disorders, are of scientific interest. One of the factors potentially associated with disease coping and QoL is child maltreatment, which is associated with a various number of negative outcomes in adulthood. Child maltreatment is defined as abuse and neglect experienced by children through their parents or other caretakers potentially causing them harm. It is divided into five subtypes of sexual abuse, physical abuse, and neglect as well as emotional abuse and neglect (11). Global prevalence rates for subtypes of child maltreatment are being reported as 12.7% for sexual abuse, 22.6% for physical abuse, 36.3% for emotional abuse, 16.3% for physical neglect, and 18.4% for emotional neglect (12) as well as about one third of a German representative population sample experiencing at least one type of child maltreatment (13). The impact of child maltreatment on developing mental health issues was considered by various studies. Experiencing one or multiple types of child maltreatment is associated with a higher risk for developing depression (14–16), anxiety and eating disorders (14) as well as personality disorders (17–19). In particular, the association between borderline personality disorder and childhood maltreatment is reported in current literature (20), for instance the earlier onset of the disorder in adults who experienced maltreatment through childhood in comparison to those who did not report maltreatment experiences (21). Furthermore, Marchetti et al. (22) were able to identify two personality profiles mediating the association between childhood maltreatment and borderline personality disorder. With one profile being characterized by a high tendency of self-criticism and the other of self-criticism in combination with dependency, they were able to suggest therapy addressing the specific traits, for example cognitive behavioral therapy, compassion focused therapy and schema therapy. Different forms of general health risk factors were found to be associated as well. Experiencing child maltreatment increases the risk for obesity, smoking, and risky sexual behavior (14, 23, 24) as well as alcohol abuse (16). While all of the subtypes are associated with negative health outcomes, a dose-response relationship was shown in more severe health outcomes through co-occurrence of different subtypes (15, 18, 23, 25). In addition to the aforementioned impacted factors, child maltreatment increases the risk of developing physical health issues. The experience of child maltreatment is associated with a higher risk of developing type 2 diabetes (26) with its association being partly mediated by obesity, smoking and high blood pressure (27, 28). It is related with a higher risk of developing chronic obstructive pulmonary disease (COPD) as well (26, 29) and with respiratory diseases in general (30, 31). Furthermore, a higher risk of developing cardiovascular disease is described (32) with higher risk of heart failure (25) and mortality in general (33). Decreased quality of life, higher ratings for anxiety and depression, and a lower cardiac function were found in patients with congenital heart disease who had experienced child maltreatment (34). To the best of our knowledge, this is the first study assessing child maltreatment in patients with CTEPH and the association of its intensity with measures of CTEPH severity, mental well-being and quality of life. Additionally, we assessed the prevalence of child maltreatment using the Childhood Trauma Questionnaire (CTQ) and compared it with the prevalence of the general German population.

## Methods

**Figure 1:**
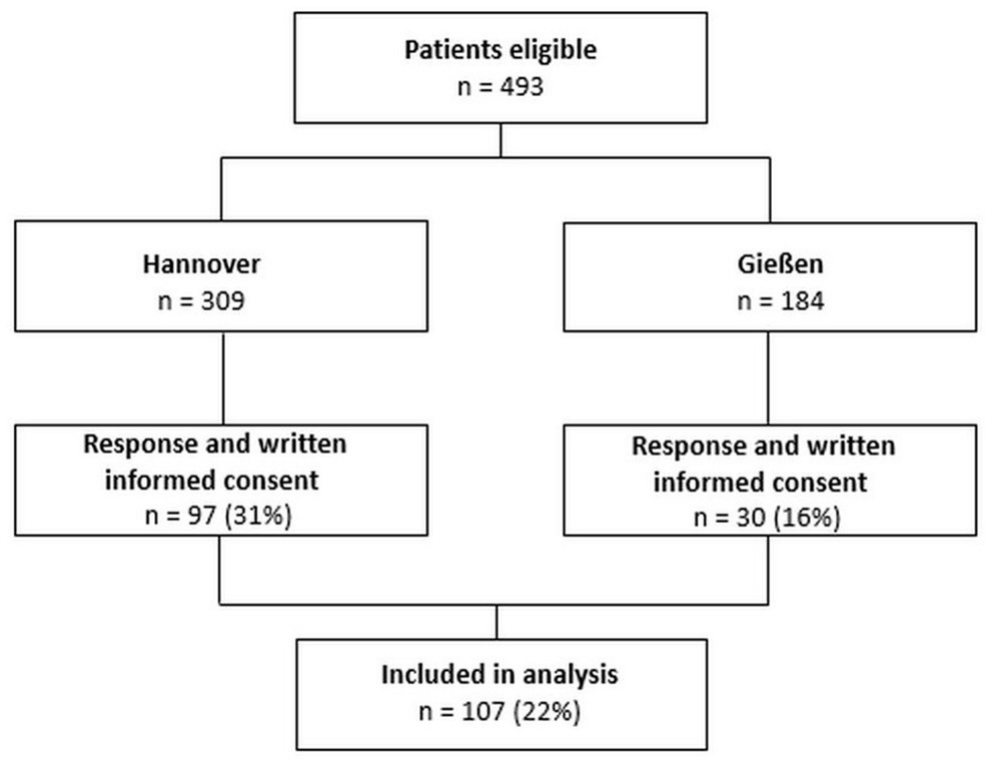
Flowchart of participation.

This cross-sectional observational study enrolled patients diagnosed with CTEPH in two German pulmonary hypertension referral centers (Hannover Medical School and University of Gießen and Marburg). Local institutional boards (Nr. 8540_BO_K_2019 for Hannover and Nr. 21119 for Giessen and Marburg) approved the study. Inclusion criteria were a diagnosis of CTEPH (WHO Group 4.1) (1, 35), age ≥ 18 years, and mental as well as physical capability of completing all questionnaires in German. Participants were excluded if they did not answer all questionnaires. A current or lifetime psychiatric diagnosis was not considered an exclusion criterium. Four hundred and ninety-three patients (309 from Hannover, 184 from Gießen) were contacted by mail after being identified for meeting the criteria from the databases of both referral centers. Recruitment took place from December 2019 until May 2021. One hundred and seven participants were recruited. All participants provided written informed consent (see Figure 1). The results presented here are part of a larger examining psychosocial factors in patients with CTEPH. We here focused on adverse childhood experiences and its impact on mental well-being and healthy lifestyle.

### Childhood Trauma Questionnaire

The Childhood Trauma Questionnaire (CTQ) (36) is a screening instrument retrospectively assessing experiences of child maltreatment including the subscales sexual abuse, physical abuse, emotional abuse, physical neglect and emotional neglect. In this study, the German short version (37, 38) was used, consisting of 28 items rated on a Likert-scale from 1 (never true) to 5 (very often true). Besides the five aforementioned subscales being measured by five items each, there are three additionally items measuring the tendency to minimize experienced maltreatment. Wingenfeld et al. (38) reported high internal consistency for emotional abuse, emotional neglect, physical abuse and sexual abuse (Cronbachs α ≥ 0.89) but not for physical neglect (Cronbachs α = 0.62). In order to measure the prevalence of each subtype, thresholds based on Walker et al. (39) were used, which were used in the prevalence study of the general German population as well (13). In accordance with Walker et al. (39), we utilized a threshold score of 10 for emotional abuse, of 15 for emotional neglect, of 8 for physical abuse, of 9 for physical neglect and of 8 for sexual abuse.

### Mental Well-Being, Quality of Life, and Lifestyle Factors

In order to measure the participants' mental well-being, the Hospital Anxiety and Depression scale (HADS) (40) was used assessing symptoms of anxiety and depression. The German version shows high internal consistency for both scales (Cronbachs α = 0.8) (41). Quality of life was investigated with the short version of the WHO Quality of Life Questionnaire (WHOQOL-BREF) (42). Internal consistency (Cronbachs Alpha) for the subscales of the German version range between α = 0.59 and α = 0.91 (43). Furthermore, anthropometric data (age, weight, height, and body mass index) as well as lifestyle factors (alcohol consumption, exercise, and smoking) and socio-demographic information (education, employment) were assessed. Exercise was defined on a scale from 1 (no sport exercise at all) to 6 (sport exercise more than three times a week). Alcohol consumption was measured in drinks per week.

### Structured Clinical Interview for DSM-5

The participants were interviewed by a trained interviewer using the structured clinical interview for DSM-5 (SCID-5; German translation) (44). The interview was conducted either face to face or *via* telephone depending on the preferences of the participants. The SCID-5 consists of a semi structured interview using questions based on the diagnostic criteria for psychiatric disorders of the Diagnostic and Statistical Manual of Mental Disorders, Fifth Edition (DSM-5) (45). The results of these interviews were used for a psychiatric characterization of the participants as reported elsewhere (46) and to control for possible confounders.

### Clinical Assessments

Body mass index (BMI), lung function tests including WHO functional class (WHO-FC), serum levels of the N-terminal fragment of pro brain natriuretic peptide (NT-proBNP), 6-min walk distance (6MWD) and the diffusion capacity of the lungs for carbon monoxide (DLCO) were used for the clinical assessments. These parameters were assessed at the time of the study while the hemodynamic parameters were assessed at time of the diagnosis.

### Statistical Analysis

In this study both, IBM SPSS Statistics 28.0 (IBM Corp., Armonk, NY, USA) and STATA 13.0 (StataCorp LP, College Station, Texas, USA) statistical software, were used for data-analysis. Depending on its appropriation, either mean and standard deviation (SD) or median and interquartile range (IQR) were used for showing continuous variables. Furthermore, and unless indicated otherwise, *n* and percent (%) are used for categorical variables. Chi-square tests were used in order to compare prevalence rates of the different types of child maltreatment in CTEPH patients and a general German population presented by Iffland and colleagues (13). Associations between general child maltreatment (total CTQ score), the child maltreatment subtypes (CTQ sub scores), mental well-being (HADS and WHOQOL), and the aforementioned lifestyle factors were analyzed performing partial correlation adjusted for age and gender. The relationship between child maltreatment (total CTQ score) and physical functioning of CTEPH patients (WHO-Functioning class) was assessed through logistic regression analysis. Mildly symptomatic patients (WHO-FC I/II) and patients with more severe symptoms (WHO-FC III/IV) were compared. *P* < 0.05 were considered statistically significant.

## Results

### Participant Characteristics

The final sample of this study consisted of 107 patients with mean age of 65 ± 14 years (range, 31–86). There was no significant sex difference between participants who experienced child maltreatment and who did not. None of the participants had current symptoms or a diagnosis of a post-traumatic stress disorder. Most patients (56%) were classified as WHO FC I/II and only 3% as WHO FC IV. For further characteristics (see Table 1).

**Table 1:** Characteristics of the patients at baseline.

	**All patients** **(*n* = 107)**	**Patients with any CTQ positive category** **(*n* = 61)**	**Patients without any CTQ positive category** **(*n* = 46)**	***p*** **-value**
Age (years)	69 (56–76)	70 (58–77)	65 (53–76)	0.066
Female sex (%)	55 (51%)	32 (53%)	23 (50%)	0.801^a^
BMI (kg/m^2^)	26 (24–31)	27 (24–32)	26 (24–30)	0.383
**Diagnosis**				
History of VTE, *N* (%)	63 (79%)	39 (87%)	24 (69%)	0.05^a^
Time since CTEPH diagnosis (years)	4 (3–7)	4 (3–8)	4 (2–7)	0.423
**WHO FC**				
I/II, *N* (%)	56 (56%)	30 (52%)	26 (60%)	0.491^a^
III, *N* (%)	42 (33%)	26 (45%)	16 (37%)	
IV, *N* (%)	3 (3%)	2 (3%)	1 (2%)	
6MWD (m)	449 (337–533)	449 (338–510)	459 (330–545)	0.462
NT-proBNP (ng/l), *N* = 86	168 (69–414)	174 (70–459)	167 (62–334)	0.728
DLCO (% pred.)	62 (53–74)	63 (51–77)	62 (55–71)	0.546
paO_2_, mmHg	62 (58–70)	64 (59–71)	61 (58–66)	0.131
**Hemodynamics at diagnosis**				
mPAP (mmHg)	42 (33–50)	41 (31–48)	42 (34–52)	0.470
PAWP (mmHg)	9 (6–12)	9 (6–11)	9 (6–12)	0.642
CI (l/min/m^2^)	2.4 (2–2.8)	2.2 (2–2.7)	2.4 (1.9–2.9)	0.602
PVR (dyn·s·cm^−5^)	506 (339–743)	480 (333–744)	509 (361–766)	0.789
CTEPH medication^*^				
No therapy	22 (28%)	8 (18%)	14 (40%)	0.07^a^
Monotherapy	47 (59%)	29 (64%)	18 (51%)	
Double combination therapy, *n* (%)	11 (14%)	8 (18%)	3 (9%)	
OAC, *n* (%)	101 (94%)	58 (95%)	43 (94%)	0.721^a^
**CTEPH interventions**				
PEA, *n* (%)	27 (25%)	15 (25%)	12 (26%)	0.860^a^
BPA, *n* (%)	37 (35%)	23 (38%)	14 (30%)	0.434^a^
BPA Sessions	2 (0–5)	2 (0–5)	2 (0–5)	0.869
**Smoking status**				
Active, *n* (%)	2 (2%)	1 (2%)	1 (2%)	0.941^a^
Former, *n* (%)	45 (42%)	25 (41%)	20 (44%)	
Never, *n* (%)	60 (56%)	35 (57%)	25 (54%)	
Packyears	12 (8–26)	9 (5–26)	18 (11–29)	0.523
**Sociodemographic items**				
Drinking (drinks per week)	0 (0–2)	0 (0–2)	0 (0–2)	0.753
Exercise Score (points)	3 (2–4)	3 (2–4)	3 (2–4)	0.509^b^
HADS-A (points)	5 (2–9)	5 (3–9)	4 (2–7)	0.190
HADS-D (points)	5 (2–8)	6 (4–9)	4 (1–6)	**0.007**
QoL-overall (points)	72 (65–85)	70 (63–78)	81 (69–92)	**0.002**
QoL-psych (points)	71 (58–79)	63 (50–79)	75 (59–86)	**0.024**
QoL-physical (points)	64 (53–79)	61 (50–73)	71 (59–82)	**0.011**

*Continuous variables are stated as median and interquartile ranges (IQR) and categorical variables are stated as n and percent (%), unless indicated otherwise. Mean and SD used because of distribution of the data*.

a *Non-parametric Pearson's chi-squared test used because of nominal scale of the variable*.

b *Non-parametric Mann–Whitney-U-Test used because of ordinal scale of the variable*.

* *There were no cases for triple combination therapy. Statistically significant values are shown as bold*.

*CM, child maltreatment; BMI, body mass index; QoL, quality of life; HADS-A, hospital anxiety and depression scale—anxiety; HADS-D, hospital anxiety and depression scale—depression; WHO FC, World Health Organization Functional Class; 6MWD, six-minute walking distance; BNP, brain natriuretic peptide; NT-proBNP, N-terminal fragment of pro-brain natriuretic peptide; DLCO, diffusion capacity of the lung for carbon monoxide; paO_2_, mmHg, arterial pO_2_; mPAP, mean pulmonary arterial pressure; PAWP, pulmonary artery wedge pressure; CI, cardiac index; PVR, pulmonary vascular resistance; IQR, inter quartile range*.

### Prevalence of Child Maltreatment in CTEPH

Fifty-seven percent of the participants had experienced at least one type of child maltreatment. 20.6% reported to have experienced emotional abuse. Furthermore, 19.6% reported physical abuse, 21.5% emotional neglect, 45.8% physical neglect, and 5.6% sexual abuse. Significantly higher rates of emotional abuse [*p* ≤ 0.001] physical abuse [*p* = 0.020] and emotional neglect [*p* = 0.028] were found compared to the general German population (13). Prevalence rates of physical neglect [*p* = 0.598] and sexual abuse did not differ [*p* = 0.791] (see Table 2).

**Table 2:** Prevalence of child maltreatment and its subtypes of CTEPH in comparison to data from the general German population.

	**CTEPH (*n* = 107)**	**General German population (Iffland et al., 2013)**	***p*** **-value**
		***n*** **= 2,500**	
Emotional abuse	22 (20.6%)	254 (10.2%)	**<0.001**
Physical abuse	21 (19.6%)	301 (12.0%)	**0.020**
Emotional neglect	23 (21.5%)	348 (13.9%)	**0.028**
Physical neglect	49 (45.8%)	1,210 (48.4%)	0.598
Sexual abuse	6 (5.6%)	156 (6.2%)	0.791

*Child maltreatment prevalence rates per group stated as percent (%), CTEPH, chronic thromboembolic pulmonary hypertension; n, total number of participants. Statistically significant values are shown as bold*.

### Association of Child Maltreatment and Mental Well-Being, Quality of Life, and Lifestyle Factors (Adjusted for age and Gender)

The total CTQ-score was correlated with anxiety symptoms of patients with CTEPH. It was negatively correlated with overall QoL and physical QoL. Emotional abuse was negatively correlated with overall QoL. Emotional neglect correlated with anxiety symptoms and showed negative correlation with physical QoL.

Physical abuse was not correlated with any aspects. Physical neglect correlated with anxiety symptoms and was negatively correlated with physical QoL. Sexual abuse was negatively correlated with overall QoL as well as physical QoL (see Table 3).

**Table 3:** Partial correlation (Spearman's *r*) of CTQ-Scores in CTEPH with HADS, QoL, Lifestyle, and WHO FC adjusted for age and gender.

	**HADS-D**	**HADS-A**	**QoL**	**Phys. QoL**	**Psych. QoL**	**BMI**	**Exercise**	**Drinks**	**Smoking**	**WHO FC**
CTQ-TS	0.240	0.369^**^	−0.324^*^	−0.383^**^	−0.250	0.130	−0.211	−0.002	0.060	0.157
CTQ-EA	0.253	0.248	−0.277^*^	−0.242	−0.253	0.201	−0.196	0.015	0.103	0.126
CTQ-EN	0.234	0.276^*^	−0.262	−0.348^**^	−0.179	0.033	−0.197	−0.024	0.143	0.146
CTQ-PA	0.206	0.266	−0.233	−0.241	−0.265	0.155	−0.117	0.043	−0.072	0.008
CTQ-PN	0.129	0.299^*^	−0.241	−0.298^*^	−0.184	0.161	−0.204	−0.014	−0.040	0.204
CTQ-SA	0.234	0.245	−0.359^**^	−0.363^**^	−0.266	0.067	−0.041	−0.081	−0.155	0.001

*CTQ-TS, childhood trauma questionnaire total score; CTQ-EA, childhood trauma questionnaire emotional abuse; CTQ-EN, childhood trauma questionnaire emotional neglect; CTQ-PA, childhood trauma questionnaire physical abuse; CTQ-PN, childhood trauma questionnaire physical neglect; CTQ-SA, childhood trauma questionnaire sexual abuse; HADS-D, hospital anxiety and depression scale—depression; HADS-A, hospital anxiety and depression scale—anxiety; QoL, WHO Quality of Life Questionnaire—total score; Phys. QoL., WHO Quality of Life Questionnaire—physical score; Psych. QoL, WHO Quality of Life Questionnaire—psychological score; BMI, body mass index; WHO FC, World Health Organization Functional Class*.

** *p < 0.01*.

* *p < 0.05*.

### Differences in the Severity of CTEPH in Association With Child Maltreatment

Using a logistic regression model to evaluate the association between child maltreatment and the severity of CTEPH at the time of the study, we defined WHO FC as depended variable with WHO FC I and II being considered asymptomatic and WHO FC III and IV being considered symptomatic. CTQ total score was defined as the independent variable. The model was statistically not significant [*p* = 0.650]. Therefore, the CTQ total score was not associated with the severity of CTEPH defined by WHO FC class.

## Discussion

In this study, we investigated the prevalence of childhood maltreatment and its association with mental well-being, quality of life, and physical health in patients with CTEPH. More than half of the patients with CTEPH had experienced at least one subtype of childhood maltreatment with physical neglect being the most frequent subtype followed by emotional neglect and emotional abuse. While the prevalence of sexual abuse and physical neglect did not differ from prevalence rates in the general German population, the prevalence of emotional abuse, emotional neglect, and physical abuse were higher in patients with CTEPH. The relatively high rates of different kinds of childhood maltreatment in CTEPH could possibly be explained by changing parenting patterns during past decades. Parenting behavior, which is now considered abusive, was considered acceptable earlier on (47). Our patients had a mean age of 65 years, were born during the 1950s and educated by parents who themselves were survivors of the second world war. Being traumatized after experiencing a war was described to contribute to the next generations' childhood trauma (48, 49). In addition to that, parenting behavior was associated with being passed on from one generation to the other referred to as intergenerational transmission, increasing the likelihood of experiencing childhood maltreatment from former maltreated parents (47). Therefore, the experienced parental trauma and the parenting behavior based on old standards may have contributed to the higher prevalence rates of child maltreatment in this cohort. The high but not significantly different prevalence of physical neglect is reported to be especially apparent in the older German population due to growing up after the Second World War and suffering from privation (50).

Overall childhood maltreatment had a negative impact on general and physical QoL and was positively associated with anxiety symptoms. Furthermore, the subtypes emotional neglect and physical neglect were positively associated with anxiety symptoms as well. Emotional abuse and sexual abuse had a negative impact on overall QoL. Emotional neglect, physical neglect, and sexual abuse had a negative impact on physical QoL. A relationship between child maltreatment and the degree of physical impairment could not be identified.

The reported negative association between childhood maltreatment and overall and physical QoL is in line with other studies (51–53). Mechanistically, it has been suggested that child maltreatment leads to biological and behavioral maladaptation, which interfere with the development of necessary skills to cope with obstacles later in life (54). The behavioral changes are associated with the development of maladaptive schemas in reaction to child maltreatment (55, 56). These schemas are hypothesized to negatively impact the way of dealing with wearing situations in adulthood, with little or no improvement over time (57). In addition to the maladaptive schemas, childhood maltreatment is characterized as increasing the risk of the disrupted ability to recognize and verbalize one's emotions, called alexithymia, increasing the risk of psychopathology (19, 58, 59). Furthermore, emotional dysregulation in general is associated with the experience of childhood maltreatment (60, 61) affecting the psychological well-being negatively by maladaptive coping with obstacles (62). The latter is explained with the need of a primary caretaker as role model in order to learn adequate emotion regulation and problem oriented coping strategies, which children, who experience childhood maltreatment, most likely do not have (63). In contrast maltreatment increases the probability of learning emotional coping strategies through the maltreating caretaker, characterized as avoidance of stressful situations as well as the suppression of emotions in general and the expression of negative emotions already during childhood and adolescence (61, 64) and later in adulthood with increased risk of engaging in substance use (65). The possible presence of maladaptive schemas, the lack of functional coping strategies with stressful events and the resulting impact on self-esteem and interaction might be responsible for the negative association between childhood maltreatment and QoL (54, 55). The association between child maltreatment and anxiety symptoms in this cohort is supported by previous research (14, 34, 66, 67). The likelihood of developing anxiety after experiencing child maltreatment might be explained through an altered stress response. Changes in stress related systems [e.g., hypothalamus-pituitary-adrenal (HPA) axis, autonomic nervous system, serotonin transporter gene] tend to increase the anxiety sensitivity (68, 69). As CTEPH is a potentially life-threatening disease and debilitating symptoms as dyspnea are present, the increased anxiety sensitivity might be considered as a reason for the associations found in this study.

We did not find an association between child maltreatment of any kind and depressive symptoms as well as with other health risk factors such as BMI, smoking, low exercise and alcohol consumption. The result contrasts with the high prevalence rates of maltreatment found in this cohort and the strong association described between the aforementioned factors and child maltreatment in other cohorts (14, 16, 23, 54, 70). Additionally, we did not find an association between physical abuse and QoL, which is in contrast to other studies pointing out the influence of physical abuse on QoL (54). The lacking associations might be explained by the low number of participants in our study, masking the effects of childhood maltreatment on psychological and behavioral factors. A further possible explanation could be resilience as it was reported to lower the risk of developing depression after the experience of child maltreatment (71). Resilience is defined as adaptation to adverse experiences, showing at least average functioning in various domains and an absence of pathology (72). It is viewed as a dynamic process which therefore does not exclude the possibility of functioning in some domains while not in others (73). The absence of the association with common health risk factors and depressive symptoms in this study might be explainable by enough protective factors within the participants to cope in a functional way with the experienced trauma instead of engaging in maladaptive behavior as smoking, low exercise, and alcohol consumption. Further investigation on the effect of childhood maltreatment and resilience simultaneously might help to further elucidate the mechanism between resilience on the one hand and maladaptation on the other hand.

Moreover, the absence of a direct impact of child maltreatment on the severity of CTEPH might be explainable with resilience as well as the underlying mechanisms and risk factors for developing CTEPH. Wegman and Stetler (31) described the association of child maltreatment with cardiovascular and respiratory diseases with a greater risk to engage in health risking behavior as smoking and consuming alcohol. Other studies described this mediating effect as well (25, 26) which could be missing in this cohort due to the lack of association between childhood maltreatment and the aforementioned health risk behaviors. Another reason for the missing impact of childhood maltreatment on CTEPH severity might refer to the dose-response relationship. Studies who investigated on the mental and physical health outcome of participants who experienced child maltreatment reported an increased risk especially in association with the co-occurrence of different subtypes (15, 23, 25, 26). In contrast to that, more than half of the participants who reported child maltreatment in this study, had experienced not more than one subtype, and less than one third had experienced three or more subtypes. Further studies with a larger sample might be needed to further investigate on the relationship between child maltreatment and severity of CTEPH.

In comparison to the general German population, three out of five maltreatment subtypes were present with higher prevalence in our CTEPH cohort. This implies the question whether childhood maltreatment might be associated with the development of CTEPH later in life. A possible link between childhood maltreatment and thromboembolic events might be seen *via* alterations of the inflammatory response. In fact, inflammation is linked to the development of CTEPH besides other risk factors such as acute pulmonary embolism, medical interventions (e.g., splenectomy and ventriculoarterial shunt) and plasmatic factors (74). Inflammatory markers like the C-reactive protein (CRP) were shown to be increased in comparison to healthy controls (75). As CRP was found to be associated with childhood trauma as well (76), changes in the inflammatory response could mediate the effect between child maltreatment and developing CTEPH. Molecular changes in maltreated individuals are associated with epigenetic changes in response to the experienced trauma. Alterations in DNA methylation are associated with altered gene expression, influencing for example the stress response and inflammatory processes (77, 78). Further studies on the mediating effect of epigenetic alterations due to child maltreatment associating it with CTEPH patients may be needed to clarify the exact relationship.

Regarding the conception of this study, limitations have to be acknowledged. Child maltreatment was assessed with the CTQ, which is based on self-report and retrospection making a recall bias possible (13, 79). Furthermore, out of the contacted CTEPH patients, only 22% responded and were included into the assessment. Hence, the sample size was low in comparison to other studies focusing on the consequences of childhood maltreatment [e.g., (25, 33)]. One contributor to the low response rate may have been the higher age of our patient population compared to other studies. Furthermore, the motivation to participate might have been limited as many of the patients were no longer followed at their PH center (especially those who had undergone successful PEA surgery). In addition, the questionnaires had been sent out without personal advance notice from their PH physicians.

In conclusion, more than half of our patients with CTEPH had experienced at least one type of child maltreatment. A history of child maltreatment had a negative impact on QoL and was associated with anxiety symptoms. In order to improve the treatment of patients with CTEPH, the presence of experienced childhood maltreatment should be considered. Further studies are needed to assess tools that may help patients to cope better with the consequences of CTEPH.

## Data Availability Statement

The raw data supporting the conclusions of this article will be made available by the authors, without undue reservation.

## Ethics Statement

The studies involving human participants were reviewed and approved by Ethics Committee, Hannover Medical School, Hannover Germany and Ethics Committee, University Medical Center Gießen und Marburg, Marburg, Germany. The patients/participants provided their written informed consent to participate in this study.

## Author Contributions

NL, JF, MH, KK, KO, and M-RD were responsible for study design, implementation of the study, statistical analysis, data interpretation, and drafting the manuscript. NL and M-RD were responsible for conducting the interviews. NL, JF, and M-RD did data collection. JK, MR, and HGa implementation of the study. IH, D-HP, HGh, DE, JK, MR, HGa, TM, and H-DK were responsible for study design, data interpretation, and revising the manuscript. All authors contributed to the article and approved the submitted version.

## Funding

This work was funded by the German Center for Lung Research (DZL).

## Conflict of Interest

MH has received honoraria for lectures and/or consultations from Acceleron, Actelion, Bayer, GSK, Janssen, MSD and Pfizer, and all outside the present study. D-HP has received honoraria for lectures and/or consultationsf rom Janssen. HGa has received personal fees from Actelion, personal fees from AstraZeneca, personal fees from Bayer, personal fees from BMS, personal fees from GSK, personal fees from Janssen-Cilag, personal fees from Lilly, personal fees from MSD, personal fees from Novartis, personal fees from OMT, personal fees from Pfizer, personal fees from United Therapeutics, outside the submitted work. HGh has received fees from Actelion, Bayer, Gilead, GSK, MSD, Pfizer and United Therapeutics, outside the present work. KK has received honoraria forconsultations and/or lectures from Eli Lilly, Janssen, Lundbeck, Neuraxpharm, Otsuka, Pfizer, Servier, Schwabe, Takeda and Trommsdorff/Ferrer, Alexion, and CannaXan (advisory board). KO has received honoraria for lectures and/or consultations from Acceleron, Actelion, Bayer, GSK, Janssen, MSD, United Therapeutics and Pfizer, and all outside the present study. H-DK was employed by Pulmonale Hypertonie eV. The remaining authors declare that the research was conducted in the absence of any commercial or financial relationships that could be construed as a potential conflict of interest.

## Publisher's Note

All claims expressed in this article are solely those of the authors and do not necessarily represent those of their affiliated organizations, or those of the publisher, the editors and the reviewers. Any product that may be evaluated in this article, or claim that may be made by its manufacturer, is not guaranteed or endorsed by the publisher.
